# Impaired spatial working memory but spared spatial reference memory following functional loss of NMDA receptors in the dentate gyrus

**DOI:** 10.1111/j.1460-9568.2007.05312.x

**Published:** 2007-02

**Authors:** B Niewoehner, F N Single, Ø Hvalby, V Jensen, S Meyer zum Alten Borgloh, P H Seeburg, J N P Rawlins, R Sprengel, D M Bannerman

**Affiliations:** 1 Department of Experimental Psychology, University of OxfordSouth Parks Road, Oxford OX1 3UD, UK; 2 Max-Planck Institute for Medical Research, Department of Molecular NeurobiologyD-69120 Heidelberg, Germany; 3 Molecular Neurobiology Research Group, Institute of Basic Medical Sciences, University of OsloN-0317 Oslo, Norway

**Keywords:** hippocampus, long-term potentiation, mouse, NR1, radial maze, synaptic plasticity

## Abstract

Novel spatially restricted genetic manipulations can be used to assess contributions made by synaptic plasticity to learning and memory, not just selectively within the hippocampus, but even within specific hippocampal subfields. Here we generated genetically modified mice (*NR1*^*ΔDG*^ mice) exhibiting complete loss of the NR1 subunit of the *N*-methyl-d-aspartate receptor specifically in the granule cells of the dentate gyrus. There was no evidence of any reduction in NR1 subunit levels in any of the other hippocampal subfields, or elsewhere in the brain. *NR1*^*ΔDG*^ mice displayed severely impaired long-term potentiation (LTP) in both medial and lateral perforant path inputs to the dentate gyrus, whereas LTP was unchanged in CA3-to-CA1 cell synapses in hippocampal slices. Behavioural assessment of *NR1*^*ΔDG*^ mice revealed a spatial working memory impairment on a three-from-six radial arm maze task despite normal hippocampus-dependent spatial reference memory acquisition and performance of the same task. This behavioural phenotype resembles that of *NR1*^*ΔCA3*^ mice but differs from that of *NR1*^*ΔCA1*^ mice which do show a spatial reference memory deficit, consistent with the idea of subfield-specific contributions to hippocampal information processing. Furthermore, this pattern of selective functional loss and sparing is the same as previously observed with the global GluR-A l-α-amino-3-hydroxy-5-methyl-4-isoxazelopropionate receptor subunit knockout, a mutation which blocks the expression of hippocampal LTP. The present results show that dissociations between spatial working memory and spatial reference memory can be induced by disrupting synaptic plasticity specifically and exclusively within the dentate gyrus subfield of the hippocampal formation.

## Introduction

Lesion studies have repeatedly implicated the hippocampus in spatial learning (O'Keefe & Nadel, 1978; Morris *et al*., 1982). The dentate gyrus (DG) is a key input node for the hippocampal formation. Perforant path fibres originating in entorhinal cortex provide a major source of highly processed sensory information to DG granule cells. Selective, fibre-sparing, colchicine lesions of the DG, like complete hippocampal lesions, result in robust impairments of both spatial working memory (SWM) and spatial reference memory (SRM; Sutherland *et al*., 1983; McNaughton *et al*., 1989; Xavier *et al*., 1999). For example, DG lesions dramatically increase both SRM and SWM errors on a four-from-eight radial maze task (Jeltsch *et al*., 2001).

*N*-methyl-d-aspartate receptor (NMDAR)-dependent long-term potentiation (LTP) is an experimental model of synaptic plasticity and is widely hypothesized to be the neural mechanism that underlies hippocampus-dependent spatial memory (Martin *et al*., 2000). Early evidence supporting this hypothesis came from pharmacological studies. Intracerebroventricular (i.c.v.) infusion of the NMDA antagonist AP5 impaired acquisition of the fixed location, hidden platform, SRM watermaze task and blocked LTP at perforant path–granule cell synapses in the DG (Morris *et al*., 1986). Indeed, the behavioural impairment was strongly correlated with the magnitude of LTP blockade in the DG measured *in vivo* in the same animals that had undergone watermaze testing (Davis *et al*., 1992).

Concerns have nonetheless been raised regarding the possibility that extra-hippocampal sensorimotor side-effects of the i.c.v. drug treatment might contribute to the behavioural impairment (e.g. Keith & Rudy, 1990; Cain *et al*., 1996). Furthermore, subsequent studies showed that rats treated with NMDA antagonists could acquire the SRM watermaze task, despite a lack of perforant path LTP, provided they had received prior, drug-free pre-training (Bannerman *et al*., 1995; Saucier & Cain, 1995). By contrast, NMDA antagonists produce robust and reliable SWM impairments even after task-specific pre-training (e.g. Tonkiss & Rawlins, 1991; Steele & Morris, 1999), suggesting potentially important differences between the substrates supporting SRM and SWM (Bannerman *et al*., 2006).

The development of genetically modified mice has greatly facilitated the study of LTP-like processes in memory. Novel, spatially restricted genetic manipulations now allow mutations to be targeted selectively to a specific brain region, thus permitting the contribution made by synaptic plasticity to learning to be assessed, not only selectively within the hippocampus but also within specific hippocampal subfields. We have, for the first time, generated genetically modified mice (*NR1*^*ΔDG*^ mice) exhibiting complete loss of the NR1 subunit of the NMDAR, specifically in the granule cells of the DG. We simultaneously assessed the contribution of NMDAR-dependent synaptic plasticity in perforant path–granule cell synapses to both SWM and SRM using a three-from-six radial arm maze task. We have previously used this task to demonstrate impaired SWM but intact SRM in mice lacking the GluR-A (GluR1) l-α-amino-3-hydroxy-5-methyl-4-isoxazelopropionate (AMPA) receptor subunit (Schmitt *et al*., 2003). This mutation disrupts the normal expression of hippocampal LTP in adult mice (Zamanillo *et al*., 1999; Hoffman *et al*., 2002; Jensen *et al*., 2003).

## Methods

### Generation of NR1^ΔDG^ mice

Mice with (*loxP*5171) (Lee & Saito, 1998) flanking *NR1* gene (*Grin1*) exons 11–18 (*NR1*^*2lox/2lox*^ mice) were generated by gene targeting in embryonic stem (ES) cells. The vector for *NR1* gene targeting was constructed from genomic 129/Sv mouse strain DNA with a similar strategy as the *NR1* gene targeting vector used previously (Single *et al*., 2000). One *loxP*5171 element was inserted into the *Hind*III site in intron 10 of the *NR1* gene by which this site was destroyed for diagnostic purposes. A *loxP*5171 flanked PGK promoter driven neomycin phosphotransferase gene (PGK*neo*) as a positive selection marker cassette harbouring a diagnostic *Eco*RI site at its 5′ border (pLoxPneo-2.5171, modified from plasmid pLoxPneo-1) (Nagy *et al*., 1998) was inserted in-sense into an artificial *Xho*I site in intron 18 of the *NR1* gene. To enable the discrimination of the mutant allele from the wild-type allele during expression analysis at the mRNA level, four silent mutations that correspond to the homologous rat sequence (Jerecic *et al*., 2001) were introduced by PCR mutagenesis into each of four adjacent codons that encode part of the M1 segment on exon 14. For linearization, the pUC19 gene targeting vector backbone was modified to yield a unique *Not*I site at the 5′ border of the genomic construct. The final gene targeting vector (pNR1^3lox^) comprised 3.5-kb 5′ and 8-kb 3′ homologous *NR1* gene sequences relative to the selection marker cassette, of which 0.9 kb 5′ to the *loxP*5171 element in intron 10 serve as the short recombinogenic arm. About 40 µg of *Not*I linearized gene targeting vector was electroporated [Bio-Rad Gene Pulser (Hercules, CA, USA), 240 V, 500 mF, 10^7^ cells] into mouse R1 ES cells as described (Nagy *et al*., 1993) and G418 resistant cells (250 µg/mL G418) were screened for homologous recombination events using PCR with primer N1in10do5 (5′-CCCTGGCTATTCTCCCATAGG-3′) located in intron 10 5′ to the gene targeting vector sequences in intron 10 and primer N1in10LOXup2 (5′-CGAAGTTATGCAGCTTATACATTC-3′) located in intron 10 on the 5′ border of the *loxP*5171 element in intron 10, yielding a PCR product of 956 bp for the homologous recombination event. The integrity of the recombined region was verified by sequencing. The PGK*neo* selection marker was selectively removed from the *NR1*^*3lox*^ allele by transient Cre (cause of recombination enzyme)-recombinase expression upon electroporation of the recombinant ES cells with moderate amounts (10 µg) of Cre-recombinase encoding plasmid pMC-Cre-recombinase (Gu *et al*., 1993). The desired removal was identified by PCR with primer N1ex18do1 (5′-CTGGGACTCAGCTGTGCTGG-3′) located on exon 18 5′ to the *loxP*5171 element in intron 18 and primer N1in18up1 (5′-AGGGGAGGCAACACTGTGGAC-3′) located in intron 18 3′ to the *loxP*5171 element in intron 18, yielding PCR products of 455 bp for the wild-type *NR1* allele and 500 bp for the mutant *NR1*^*2lox*^ allele. Successful homologous recombination and PGK*neo* cassette removal was further verified via *Eco*RI Southern blot analysis using a 830-bp *Avr*II-*Eco*RV rat *NR1* cDNA fragment (Single *et al*., 2000) as a probe. Correctly targeted ES cells were injected into mouse blastocysts (C57Bl/6) and the resulting chimeric animals were bred with C57Bl/6 mice leading to positive heterozygous offspring with a mendelian distribution of the *NR1*^*2lox*^ allele. The *NR1*^*2lox*^ allele was monitored via PCR genotyping of genomic DNA from tail tip biopsies using primers N1ex18do 1 and N1in18up1 (see above). Mice homozygous for the targeted *NR1* allele were called *NR1*^*2lox*^*.* Expression levels of the mutant *NR1*^*2lox*^ allele and the wild-type allele were comparable in heterozygous mice as estimated from the peak heights of the four discriminative silent mutations on exon 14 in the sequence chromatograms of RT-PCR products. Moreover, Western blot analysis of forebrain protein revealed no difference in *NR1* subunit expression in C57Bl/6 mice and *NR1*^*2lox*^ mice.

**Fig. 1:**
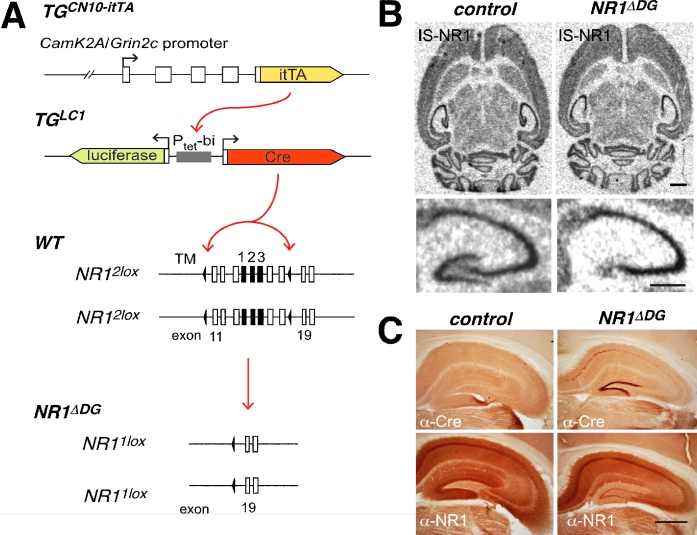
Depletion of functional NMDAR in *NR1*^*ΔDG*^ mice. (A) Genetic elements used for cell-specific *NR1* gene deletion. Expression of itTA from the *CamK2A/Grin2c* hybrid promoter (transgene from line *TG*^*CN10–itTA*^) drives Cre-recombinase expression by activation of the bidirectional Ptet_bi_ promoter of the luciferase/Cre tet-responder (transgene from line *TG*^*LC1*^). Cre-recombinase excises the *loxP* (black triangle) flanked exons 11–18 (boxes) of the gene-targeted modified *NR1*^*2lox*^ alleles. Exons encoding membrane regions 1–3 (TM1–3) are given in black. In cells with active Cre the *NR1*^*2lox*^ alleles are converted to *NR1*^*1lox*^ alleles. (B) *In situ* hybridization of littermate control animals (left) and DG-specific *NR1* deletion mice (*NR1*^*ΔDG*^) (right) with *NR1*-specific probe. The lower panels give a zoom of the hippocampus from the respective upper panels (scale bars: 1 mm). (C) Cre immunohistochemistry (top panels) of littermate *NR1*^*2lox*^ (control, left) and *NR1*^*ΔDG*^ mice (right) and with anti-NMDAR1 antibody (bottom panels) (scale bar: 1 mm).

To inactivate functionally the floxed *NR1*^*2lox*^ alleles by Cre recombinase specifically in DG granule cells, we employed *NR1*^*2lox*^ mice (Shimshek *et al*., 2006) and the transgenes from transgenic mouse lines *TG*^*CN10–itTA*^ and *TG*^*LC1*^(Fig. 1A). Mice from the line *TG*^*LC1*^ encode a bidirectional tTA-dependent responder gene cassette containing tTA responsive minigenes for Cre recombinase and the firefly luciferase (Schonig *et al*., 2002). Mice from mouse line *TG*^*CN10–itTA*^ contain a tTA transgene, which is specifically expressed in DG granular cells and some CA1 pyramidal neurons. The Cre-recombinase expression and Cre-recombinase activity patterns of *TG*^*CN10–itTA*^ and *TG*^*LC1*^ have been described previously (Krestel *et al*., 2004). For the histological, electrophysiological and behavioural analysis of DG-specific NMDAR depletion we produced sizeable cohorts of *NR1*^*2lox*^ mice transgenic for transgenes of *TG*^*CN10–itTA*^ and *TG*^*LC1*^ (*NR1*^*ΔDG*^ mice), along with *NR1*^*2lox*^ littermates lacking the transgenes of either *TG*^*CN10–itTA*^ or *TG^LC1^* referred to herein as control mice.

Molecular and biochemical experiments with mice were performed according to the institutional guidelines at the animal facility of the Max Planck Institute for Medical Research. Genetic manipulations of mouse embryos were licensed by the ‘Regierungspräsidium Karlsruhe’ (37-9185.81/35/97).

### In situ *hybridization*

*In situ* hybridizations were performed as described previously (Jerecic *et al*., 2001). Mice were anaesthetized with CO_2_ and killed by cervical dislocation; the brains were removed and frozen on dry ice immediately. Horizontal 15 µm cryostat sections were mounted on slides and antisense oligonucleotide NR1ratis (5′-GAACTGACAGTCCTACTAGCAACCACAGTGTGCTC-3′) was used for monitoring the expression of the *NR1*^*2lox*^ allele. The oligonucleotides were 3′-endlabelled with terminal deoxynucleotide transferase and [α-^35^S]-dATP. Cryostat sections were hybridized overnight at 42 °C in 50% formamide, 0.6 m NaCl, 0.06 m sodium citrate (4 × SSC), 10% dextran sulphate with 1 pg/mL probe. Sections were washed in 1 × SSC at 60 °C for 20 min and exposed to Kodak XAR-5 films (Sigma-Aldrich Chemie, Steinheim, Germany).

### Immunohistochemistry

Immunohistochemistry was performed as described previously (Jerecic *et al*., 2001; Krestel *et al*., 2004). In brief, coronal sections (100 µm) were stained with primary antibodies anti-NMDAR1 (1 : 2000 polyclonal, Chemicon, Temecula, CA, USA), anti-Cre (1 : 8000, polyclonal, BabCO, Berkley, CA, USA), and peroxidase coupled secondary antibodies (1 : 600, Vector, Burlingame, CA, USA). Antibody-treated brain slices were stained with DAB (Sigma-Aldrich Chemie). DAB-developed slices were then mounted on slides, air-dried, covered with cover slips using eu-kitt (O. Kindler, Freiburg, Germany) and finally imaged with a Zeiss microscope.

### Electrophysiological assessment of NR1^ΔDG^ mice

Experiments were performed on hippocampal slices prepared from adult (2–4 months old) *NR1^Δ^*^*DG*^ mice and wild-type control mice. The animals were killed with an overdose of desflurane anaesthetic (Suprane, Baxter AS, Oslo, Norway) and the brains were removed and cooled in artificial cerebrospinal fluid (ACSF, 0–4 °C, bubbled with 95% O_2_/5% CO_2_, pH 7.4) containing (in mm): 124 NaCl, 2 KCl, 1.25 KH_2_PO_4_, 2 MgSO_4_, 1 CaCl_2_, 26 NaHCO_3_ and 12 glucose. Transverse slices (400 µm) were cut from the middle portion of each hippocampus with a vibroslicer and placed in a humidified interface chamber at 30 ± 1 °C and perfused with ACSF containing 2 mm CaCl_2_. In order to enhance the induction of LTP in the DG, we partially blocked GABA_A_-mediated inhibition with (–)-bicuculline methochloride (6 µm; Tocris Cookson Ltd, Bristol, UK). The resulting hyperexcitability was counteracted by increasing the concentration of Ca^2+^ and Mg^2+^ to 4 mm in accordance with earlier reports (Wigström & Gustafsson, 1983, 1985). In some experiments 50 µm dl-2-amino-5-phosphonopentanoic acid (DL-AP5; Sigma-Aldrich, Oslo, Norway) was present during the experiments in order to block NMDAR-mediated synaptic plasticity (see Supplementary material).

Orthodromic synaptic stimuli (50 µs, < 300 µA, 0.1 Hz) were delivered alternately through two tungsten electrodes, either with one situated in the stratum radiatum and another in the stratum oriens of the CA1 region, or with electrodes in the outer and middle molecular layer of the upper blade of the dentate area. Extracellular synaptic responses were monitored by two glass electrodes (filled with ACSF) placed in the corresponding synaptic layers. After obtaining stable synaptic responses in both pathways (0.1 Hz stimulation) for at least 10–15 min, one pathway was tetanized (100 Hz, 1 s) while the other served as a non-tetanized control pathway. The tetanic stimulation strength was just above the threshold for generation of a population spike in response to a single test stimulus.

Synaptic efficacy was assessed by measuring the slope of the field EPSP (fEPSP) in the middle third of its rising phase. Six consecutive responses (1 min) were averaged and normalized to the mean value recorded 1–4 min prior to tetanic stimulation. Data were pooled across animals of the same genotype and are presented as mean ± SEM. The difference between tetanized and non-tetanized pathways was statistically evaluated by a Student's paired, two-tailed *t*-test, and when comparing LTP levels between control and *NR1*^*ΔDG*^ mice we used a linear mixed model analysis.

Electrophysiological experiments were conducted according to the Norwegian Animal Welfare Act and the European Union's Directive 86/609/EEC. Efforts were made to minimize the number of animals used.

### Behavioural assessment of NR1^ΔDG^ mice

#### Subjects

All behavioural testing was conducted with experimentally naïve, age-matched, male transgenic mice and littermates. The cohort consisted of *NR1*^*ΔDG*^ mice (*n* = 8), heterozygous *NR1*^*2lox*^/*TG*^*LC1*^ mice (*n* = 12) and heterozygous *NR1*^*2lox*^/*TG*^*CN10–itTA*^ mice (*n* = 4). As the behavioural performance of the two groups of heterozygous mice was indistinguishable, their data were combined forming a single control group (control; *n* = 16). All mice were first subjected to a battery of tests assessing sensorimotor function and emotionality (see Supplementary material).

#### Assessment of spatial memory on the radial maze

Spatial memory was assessed using a six-arm radial maze which was made of wood and painted grey (Schmitt *et al*., 2003). Each arm (60 × 7 cm) was surrounded by a 1 cm raised edge and extended from a circular central platform (18 cm diameter). At the end of each arm was a stainless steel food well. Mice were rewarded with 0.1 mL sweetened, condensed milk (diluted 50 : 50 with water). The maze was elevated 80 cm above the floor in a well-lit laboratory (6.3 × 2.7 m) which contained various extra-maze cues (e.g. laboratory equipment, stools, bench, posters). The central platform was surrounded by a transparent Perspex cylinder (18 cm diameter, 30 cm high). At the entrance to each arm of the maze was a Perspex door (6 cm wide, 7 cm high) which could be controlled by the experimenter using a series of strings.

Mice were maintained on a restricted feeding schedule at 85% of their free-feeding weights. The mice were first habituated to drinking sweetened, condensed milk on two arms of an elevated Y-maze (Reisel *et al*., 2002) in their colony holding room (i.e. not the testing room). Once all the mice were running freely on the Y-maze and readily consuming the milk rewards, testing on the radial arm maze began.

#### Spatial reference memory acquisition

Mice were first trained to discriminate between baited and non-baited arms on a radial maze task in which the same three out of six arms were always baited. The three baited arms were allocated such that two of these arms were adjacent and the third was between two non-rewarded arms (e.g. arms 1, 2 and 4). Different combinations of arms were used as far as possible, although the arm allocations were counterbalanced across groups. At the start of a trial, a mouse was placed individually on the central platform. Mice were allowed to explore freely and consume all the milk rewards available. During this acquisition phase, Perspex doors prevented mice from re-entering an arm that they had already visited on that trial (Schmitt *et al*., 2003). All the doors were closed each time the mouse returned to the central platform, and confined the mouse there for 5 s until the next choice. Once an arm had been visited, its door remained closed for subsequent choices. Thus, all six doors were open for the first choice, five for the second choice, four for the third choice, and so on. Using this testing procedure it was not possible for the mice to make working memory errors. This provides a pure test of SRM acquisition, and is dependent upon the hippocampus (Schmitt *et al*., 2003). SRM errors were defined as entries into arms that were never baited (maximum of three errors per trial). The maze was rotated periodically to prevent the mice from using intra-maze cues to solve the task. Mice received 32 trials in total. Data were arranged in eight blocks of four trials for analysis. By this stage all of the animals had acquired the SRM component of the task and were making very few, if any, errors.

#### Simultaneous assessment of spatial working and reference memory

The SWM component of the task was then introduced. The mice received a further 24 trials (with an inter-choice interval of 5 s) in which the same three out of six arms were baited, but now they were no longer prevented from re-entering a previously chosen arm. The doors were solely used to retain the animals on the central platform between choices. SWM errors were scored when a mouse entered an arm that had already been visited on that trial. SRM errors were scored as before. The effect of increasing the retention interval between successive choices was then assessed (Tonkiss & Rawlins, 1991; Steele & Morris, 1999; Lee & Kesner, 2002). The minimum amount of time that the animal spent on the central platform between choices with all doors closed was increased from 5 to 15 s and a further 24 trials were conducted.

#### Data analysis

Each type of memory error (SRM and SWM) was analysed separately. The data were analysed in blocks of four trials. Where the assumptions of normality and equal variance were met, data were analysed by anova with subsequent analysis of simple main effects where appropriate. If the data failed to satisfy these assumptions, transformations (square root transform) were applied and anova performed on the transformed data set. To make the figures more legible, however, all the data are presented as un-transformed means (± SEM).

#### Assessment of pattern separation

We then further analysed the data to determine whether the likelihood of errors made by the mice was dependent on the spatial separation of the arms that were to be discriminated. Computational models have allocated specific roles in information processing to the different subfields of the hippocampal formation, and have suggested a key role for the DG in spatial pattern separation (Marr, 1971; McNaughton, 1989; O'Reilly & McClelland, 1994; Shapiro & Olton, 1994; Rolls, 1996; Rolls & Treves, 1998; Gilbert *et al*., 2001). For each animal the three allocated baited arms consisted of two adjacent baited arms and one baited arm that was bordered on either side by non-baited arms (e.g. arms 1, 2 and 4 or 3, 4 and 6, etc.). If the *NR1*^*ΔDG*^ mice exhibit impaired pattern separation then it might be expected that they will display (i) more SRM errors during acquisition into a non-rewarded arm bordered by two rewarded arms than into adjacent non-rewarded arms (having adjusted for the 2 : 1 ratio of adjacent to single arms), and (ii) more SWM errors into adjacent baited arms compared with repeat entries into the single baited arm (again having adjusted for the 2 : 1 ratio of adjacent to single arms). Presumably two adjacent arms have greater overlap between their relevant spatial cues than two arms that are separated by at least one other arm; hence discriminating between adjacent arms entails a greater need for spatial pattern separation. We therefore separately examined the nature of the spatial memory errors made during (i) SRM acquisition and (ii) testing on the SWM component of the task at both the 5-s and 15-s delay conditions (making allowance for the 2 : 1 ratio of adjacent to single arms within each condition). Errors into the single arm and errors into the adjacent arms (divided by 2) were thus compared for each phase of the study.

Behavioural experiments were conducted under the auspices of the UK Home Office Project and Personal licenses held by the authors (UK).

## Results

### Generation of NR1^ΔDG^ mice

We generated mice carrying floxed *NR1* alleles and used tTA-induced Cre-recombinase expression to destroy the *NR1* gene specifically in DG cells (Fig. 1A; see also Krestel *et al*., 2004). Analysis of brains from several postnatal day 60 mice by *in situ* hybridization (Fig. 1B) and immunocytochemistry (Fig. 1C) revealed that in all *NR1*^Δ*DG*^ mice, the NR1 mRNA and NR1 subunits were selectively reduced in DG granule cells, whereas mRNA and protein levels appeared unchanged in other subfields of the hippocampal formation, as well as in other forebrain structures.

### Electrophysiological assessment of NR1^ΔDG^ mice

**Fig. 2:**
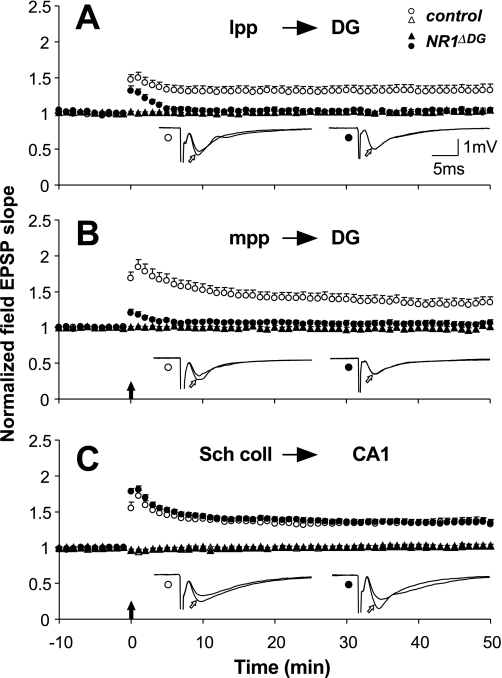
Specific loss of LTP in DG granule cell synapses in *NR1*^*ΔDG*^ mice. Summary graphs of normalized field EPSP slopes evoked in the control (open circles) and the *NR1*^*ΔDG*^ mice (filled circles) in the lateral perforant path (A), medial perforant path (B), and in the CA1 region (C) For the sake of clarity, only the non-tetanized pathways of *NR1*^*ΔDG*^ mice are shown in A and B (filled triangles), whereas the non-tetanized pathways (filled and open triangles) for both groups of animals are shown in C. The insets show superimposed means of six consecutive synaptic responses in the tetanized pathway before and 40 min after (open arrows) tetanization from control (left) and *NR1*^*ΔDG*^ mice (right). Filled arrows indicate the time of tetanic stimulation. Vertical bars indicate SEM.

Deletion of NR1 subunit expression in DG cells led to a complete loss of NMDAR-mediated LTP at DG synapses. Tetanization of the afferent fibres in slices from six control mice produced a persistent homosynaptic potentiation observed 40–45 min after the tetanic stimulation in both the lateral perforant path/granule cell synapses (tetanized vs. non-tetanized pathway: 1.30 ± 0.08 vs. 1.00 ± 0.03, *P* < 0.01, *n* = 16; Fig. 2A) and in the medial perforant path/granule cell synapses (1.32 ± 0.08 vs. 0.98 ± 0.03, *P* < 0.01, *n* = 19; Fig. 2B). In slices from seven *NR1*^*ΔDG*^ mice, however, the tetanized pathway was not significantly different from the non-tetanized pathway, either in the lateral perforant path pathway (1.03 ± 0.03 vs. 1.02 ± 0.03, *n* = 19, *P* = 0.77; Fig. 2A) or in the medial perforant path pathway (1.06 ± 0.04 vs. 0.99 ± 0.03, *n* = 18, *P* = 0.12; Fig. 2B), demonstrating that in both pathways LTP is NMDAR-dependent (Hanse & Gustafsson, 1992), and is lost when the expression of NMDARs in DG granule cells is abolished by Cre-recombinase mediated *NR1* gene destruction.

Despite the fact that we could not detect a loss of the NR1 subunit mRNA or NR1 protein in other principal neurons in the hippocampal formation, we found that Cre recombinase was expressed in some CA1 cells (Fig. 1C). In addition, we found that the DG-specific Cre expression model showed some Cre recombinase activity in CA1 pyramidal cells (Krestel *et al*., 2004). To investigate if this residual Cre activity affects NMDAR function in CA1 we analysed the NMDAR-dependent LTP at Schaffer collateral–CA1 synapses (Collingridge *et al*., 1983). Statistical evaluation revealed no difference in the amount of LTP obtained in the two genotypes (*P =* 0.96; Fig. 2C). The average fEPSP slope in control mice measured 1.35 ± 0.03 (*n* = 23) of the pre-tetanic value 40–45 min after tetanization (non-tetanized pathway: 1.02 ± 0.03), and in *NR1*^*ΔDG*^ mice the magnitude of LTP was 1.34 ± 0.04 (*n* = 26) (non-tetanized pathway: 1.02 ± 0.01).

### Behavioural assessment of NR1^ΔDG^ mice

**Fig. 3:**
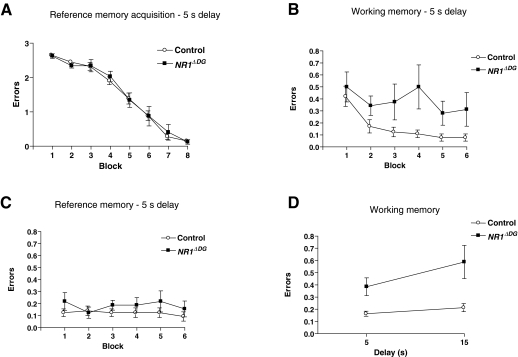
*NR1*^*ΔDG*^ mice display normal spatial reference memory but impaired spatial working memory on a 3/6 radial arm maze task. (A) Mean (± SEM) number of reference memory errors per trial (maximum of three) for control (white circles; *n* = 16) and *NR1*^*ΔDG*^ mice (black squares; *n* = 8) during reference memory acquisition in the 3/6 radial arm maze task (doors prevented working memory errors in this phase of the experiment). Each block consisted of four trials. (B) Mean (± SEM) number of working memory errors per trial during simultaneous assessment of working and reference memory performance on the task (doors no longer prevented working memory errors). The inter-choice interval was 5 s. Each block consisted of four trials. (C) Mean (± SEM) number of reference memory errors per trial during simultaneous assessment of working and reference memory performance. (D) Mean (± SEM) number of working memory errors per trial (averaged over 24 trials) during testing with an inter-choice interval of either 5 or 15 s.

Spatial learning was assessed on a radial maze. Mice were first trained to discriminate between three initially baited and three never-baited arms under conditions in which it was not possible to make working memory errors (Schmitt *et al*., 2003). Both control and *NR1*^*ΔDG*^ mice successfully acquired the 3/6 SRM task, making progressively fewer errors as training proceeded (Fig. 3A; main effect of block, *F*_7,154_ = 133.1, *P* < 0.0001). There was no evidence of any SRM impairment in the *NR1*^*ΔDG*^ mice (main effect of group and groups by blocks interaction, both *F* < 1).

The working memory component of the task was then introduced. *NR1*^*ΔDG*^ mice made significantly more SWM errors than control animals (main effect of group for square root transformed data, *F*_1,22_ = 11.9, *P* < 0.005; block, *F*_5,110_ = 2.9, *P* < 0.05; interaction, *F* < 1; Fig. 3B). Within these same trials, both groups of mice continued to make very few reference memory errors, and never differed on this measure (main effect of group for square root transformed data, *F*_1,22_ = 1.5, *P* > 0.20; main effect of block and groups by blocks interaction, both *F* < 1; Fig. 3C).

The effect of increasing the retention interval between successive choices from 5 to 15 s was then assessed. Both groups made more working memory errors and again the *NR1*^*ΔDG*^ mice made more errors than controls (main effect of group for square root transformed data, *F*_1,22_ = 10.0, *P* < 0.005; main effect of block, *F*_5,110_ = 10.5, *P* < 0.0001; groups by blocks interaction, *F* < 1). There were still no group differences in terms of SRM errors (main effect of group for square root transformed data, *F*_1,22_ = 1.7, *P* > 0.20; groups by blocks interaction, *F*_5,110_ = 1.4, *P* > 0.20). A comparison of working memory performance across the two delay conditions revealed a significant effect of delay condition (*F*_1,22_ = 7.4, *P* < 0.05), but no significant groups by delays interaction (*F*_1,22_ = 2.8, *P* > 0.10; Fig. 3D).

**Fig. 4:**
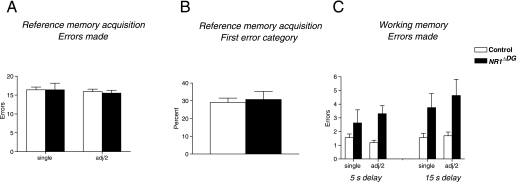
*NR1*^*ΔDG*^ mice display normal spatial pattern separation on the radial arm maze task. (A) Mean (± SEM) total number of reference memory errors during acquisition (32 trials) to the single non-baited arm and adjacent non-baited arms (divided by 2) for control (white bars; *n* = 16) and *NR1*^*ΔDG*^ mice (black bars; *n* = 8). (B) Mean (± SEM) percentage of trials during acquisition (32 trials) on which the first reference memory error is to the single non-baited arm. (C) Mean (± SEM) total number of working memory errors to the single-baited arm and adjacent baited arms (divided by 2) during testing with either a 5- or 15-s inter-choice interval (24 trials for both).

We then further analysed the data to determine whether the likelihood of errors made by the mice was dependent on the spatial separation of the arms that were to be discriminated. Summed across reference memory training, both the *NR1*^*ΔDG*^ and the control mice were almost exactly as likely to make an error to a single as to an adjacent non-rewarded arm (mean errors for *NR1*^*ΔDG*^ mice, 16.38 to the single arm and 15.5 per adjacent arm; mean errors for the control mice, 16.38 per single arm and 15.91 per adjacent arm; *F* < 1; Fig. 4A). A separate analysis considered whether the first reference memory error made in each testing trial was more likely to be to the single, never-baited arm or one of the adjacent, never-baited arms, over each of the eight, four-trial blocks of acquisition. Again, there were no differences in error patterns between groups (main effect, *F* < 1; interaction with blocks of training, *F*_7,154_ = 1.21, *P* > 0.20; Fig. 4B), even though during training overall error probabilities changed from high to low levels in both groups.

In contrast to reference memory acquisition, which was normal in *NR1*^*ΔDG*^ mice, overall working memory performance had differed between groups. We therefore considered whether this difference might result from impaired spatial pattern separation. Although working memory errors were more common overall in the *NR1*^*ΔDG*^ mice, they were no more likely to be into adjacent than into single initially baited arms (Fig. 4C). anova revealed no overall main effect of error type (*F <* 1), and importantly no groups by error type interaction (*F*_1,22_ = 1.7, *P* > 0.20) nor any groups by error type by delay condition interaction (*F <* 1). Analysis of simple main effects showed that the *NR1*^*ΔDG*^ mice made significantly more of both error types (*F*_1,37_ > 6.1, *P* < 0.02 for both), and that there were no differences in the number of each error type made by either group (both *F* < 1). Inspection of the data revealed that neither wild-type nor *NR1*^*ΔDG*^ mice were visiting the baited arms in a consistent order across trials.

## Discussion

In the present study we generated genetically modified mice (*NR1*^*ΔDG*^ mice) exhibiting substantial loss of the NR1 subunit of the NMDAR specifically in the granule cells of the DG. There was no evidence of any reduction in NR1 subunit levels in any of the other hippocampal subfields, or anywhere else in the brain. *NR1*^*ΔDG*^ mice displayed severely impaired LTP in both medial and lateral perforant path inputs to the DG, whereas LTP was unchanged in CA3-to-CA1 cell synapses in hippocampal slices. Behavioural assessment of the *NR1*^*ΔDG*^ mice revealed a clear SWM impairment in the absence of any effect on hippocampus-dependent SRM performance on the same task. Importantly, this contrasts with the effects of fibre-sparing, colchicine lesions which disrupt both SWM and SRM performance (Sutherland *et al*., 1983; McNaughton *et al*., 1989; Xavier *et al*., 1999; Jeltsch *et al*., 2001). The present results therefore demonstrate that SWM and SRM are, at least in part, subserved by different information processing mechanisms within the hippocampus.

The working memory deficit in the *NR1*^*ΔDG*^ mice is unlikely to result from non-mnemonic effects of the mutation on sensorimotor or motivational aspects of performance, because these same animals showed normal SRM acquisition and performance on the same apparatus with the same sensorimotor and motivational demands, and the same spatial cues. Moreover, a full battery of tests, including explicit assays of motor function and emotionality, revealed that in general control and *NR1*^*ΔDG*^ mice were indistinguishable (see Supplementary material). The *NR1*^*ΔDG*^ mice did, however, appear less anxious on some measures in the successive alleys test (a modified form of the elevated plus maze). This may reflect the hypothesized role of the hippocampus, particularly its ventral portion, in anxiety (Richmond *et al*., 1999; Gray & McNaughton, 2000; Kjelstrup *et al*., 2002; Bannerman *et al*., 2004; McHugh *et al*., 2004), although this reduced anxiety phenotype was not observed on the other tests of emotionality that were conducted. The absence of a sensorimotor phenotype in these mice is obviously not surprising in view of the spatially restricted nature of the mutation, but highlights the advantage of this more selective approach.

Spatially restricted genetic modifications have also allowed the role of synaptic plasticity in the other hippocampal subfields to be examined. For example, specific deletion of the NR1 subunit of the NMDAR in CA3 resulted in impaired performance on a modified version of the classic Morris watermaze task (Nakazawa *et al*., 2002). Despite successful acquisition of the standard reference memory paradigm, mutant animals performed less well than controls during a recall test that was conducted with a reduced number of the original cues. This was consistent with a deficit in pattern completion and thus with the predictions that have been made by computational models (Marr, 1971; McNaughton & Morris, 1987; Hasselmo *et al*., 1995; Rolls, 1996). In addition, in a subsequent study *NR1^Δ^*^*CA3*^ mice were also impaired in a watermaze SWM, delayed-matching to place task, after continued training to daily novel platform locations (Nakazawa *et al*., 2003). Thus, at least in some respects, the present demonstration of impaired SWM but preserved SRM in *NR1*^*ΔDG*^ mice shows some parallels with the behavioural phenotype of the *NR1*^Δ*CA3*^ mice. Both mouse lines demonstrated intact SRM acquisition and recall provided all of the spatial cues were still available, but were nonetheless impaired on a SWM task. There is thus some similarity between the phenotypes resulting from NR1 deletion in the DG and from NR1 deletion in its downstream target in CA3. In contrast to both of these mouse lines, deletion of NR1 in the CA1 subfield of the hippocampus leads to impaired SRM acquisition in the watermaze (Tsien *et al*., 1996). Although the generality of this finding to other tests of SRM such as the radial maze remains to be established, it would appear that the development of spatially restricted genetic modifications has identified specific and dissociable roles for synaptic plasticity in the different hippocampal subfields.

Computational models have also suggested a role for the DG in pattern separation (Marr, 1971; McNaughton, 1989; O'Reilly & McClelland, 1994; Shapiro & Olton, 1994; Rolls, 1996; Rolls & Treves, 1998). Empirical evidence in support of this theory has so far come from lesion studies in rats. Selective, fibre-sparing colchicine lesions of the DG affect performance on both spatial working and reference memory tasks (Sutherland *et al*., 1983; McNaughton *et al*., 1989; Xavier *et al*., 1999). More recently, DG lesions restricted to dorsal hippocampus (but not dorsal CA1 lesions) have been shown to produce deficits in spatial working memory on a matching to place task but, importantly, the impairment was only evident when the two spatial locations that were to be discriminated were close together, thus presumably maximizing the need for pattern separation (Gilbert *et al*., 2001). Further analysis of our data revealed that *NR1*^*ΔDG*^ mice made substantially and significantly more SWM errors than controls to both the single and the adjacent baited arms. They were just as likely to return in error to the single baited arm as to an adjacent baited arm. There was no evidence of a significant difference in the number of errors made to single and adjacent baited arms for the knockout mice. These results therefore fail to provide positive evidence for the hypothesis that NMDAR-mediated synaptic plasticity in the DG supports aspects of spatial pattern separation. The present results cannot, however, completely rule out a role for NMDARs in the DG in spatial pattern separation in the present task, although they do clearly show that any putative role must be restricted to the working memory component. Furthermore, if a failure of pattern separation is indeed responsible for the working memory dysfunction in *NR1*^Δ*DG*^ mice, then even arms separated by 120° must retain sufficient stimulus overlap to require spatial pattern separation. It is perhaps surprising that the change in degree of overlap between arms 60° apart and arms 120° apart was not sufficient to reveal a graded working memory impairment in errors made to single or paired arms. It nonetheless remains possible that using a radial arm maze with a greater number of arms, so that adjacent arms are even closer together and single arms could be even further apart than is possible with a six-arm maze, might have revealed a clear, graded impairment in the *NR1*^Δ*DG*^ mice as a function of degree of arm separation (but see also Gilbert *et al*., 2001).

We have also previously observed the simultaneous coexistence of impaired SWM with spared SRM performance in genetically modified mice with a global deletion of the GluR-A subunit of the AMPA receptor (Reisel *et al*., 2002), including studies which used the very same radial maze task (Schmitt *et al*., 2003, 2005). GluR-A^–/–^ mice, like the *NR1*^*ΔDG*^ mice in the present study, were perfectly able to discriminate between and remember which arms of a radial maze are baited and which are never baited (SRM), but they are unable to keep track of which arms they have entered on a particular visit to the maze, and thus avoid repeat entry (SWM) errors. On the basis of these results we have suggested that there are two distinct and dissociable information processing mechanisms within the hippocampus (Schmitt *et al*., 2004). We have suggested that one is a GluR-A-dependent system that allows the animal to respond rapidly and flexibly on the basis of trial-specific conditional information that needs to be retrieved from memory. This presumably underlies SWM performance, and could make a key contribution to aspects of episodic memory in humans. The other is a GluR-A-independent mechanism allowing the associative strength or reward valence of places or locations in the environment to be increased and/or decreased gradually or incrementally over many trials. The latter could underpin SRM acquisition on tasks such as the Morris watermaze or radial maze.

The limitation of studying global knockouts is that the deletion of the GluR-A subunit is not exclusive to the hippocampus and that it is often difficult to ascribe the behavioural phenotype to a particular brain area. Even forebrain-specific manipulations provide only a relatively limited increase in selectivity, and are not completely hippocampus-specific (Schmitt *et al*., 2005). Therefore, an alternative explanation of the impaired SWM and spared SRM in GluR-A^–/–^ mice might be that deficits in areas of the brain other than the hippocampus which mediate specific aspects of working memory performance, such as areas of the frontal lobe, underlie the behavioural effects, and that the GluR-A deletion does not in fact affect hippocampal spatial information processing, thus leaving SRM performance unaffected. Although we have previously reasoned that the absence of robust and enduring frontal lesion effects on SWM tasks such as the radial maze and the spatial non-matching to place version of the T-maze argues against such an account (e.g. Shaw & Aggleton, 1993; Aggleton *et al*., 1995; Delatour & Gisquet-Verrier, 1996, 2000; Dias & Aggleton, 2000), this is logically a weaker position than being able to demonstrate a SWM impairment, in the absence of an SRM deficit, as a result of a manipulation that is exclusively hippocampal. The present results now show that it is indeed possible to dissociate SWM from SRM on the three-from-six radial maze task with an exclusively hippocampal manipulation.

Therefore, the present results with *NR1*^*ΔDG*^ mice suggest that NMDAR-mediated synaptic plasticity in the DG contributes to a memory mechanism that encodes information associated with particular events or episodes. This deficit in *NR1*^*ΔDG*^ mice is consistent with previous pharmacological studies that have reliably shown SWM impairments following i.c.v. infusion of the selective NMDA antagonist AP5 (Tonkiss & Rawlins, 1991; Steele & Morris, 1999; Bast *et al*., 2005), at doses of the drug which have been shown also to block perforant path–granule cell LTP in the DG *in vivo* (Good & Bannerman, 1997). Furthermore, Lee & Kesner (2002) showed that infusion of AP5 directly into the DG induces delay-dependent SWM impairments. However, the present study represents a major advance over pharmacological approaches where the diffusion of the drug to other subfields and other brain structures is inevitably less well controlled.

Arguably, the present demonstration of normal SRM acquisition in the *NR1*^*ΔDG*^ mice is also potentially consistent with the previous report that AP5-treated rats are able to acquire a hippocampus-dependent SRM task in the watermaze, despite a complete block of LTP at perforant path–granule cell synapses, provided they have received prior task-specific pre-training (Bannerman *et al*., 1995). A precise explanation as to why the presence or absence of pre-training, the exact nature of the pre-training and its extent may all influence the subsequent outcome of studies assessing the effects of NMDAR antagonists on SRM remains to be fully formulated (Bannerman *et al*., 2006).

In conclusion, *NR1*^*ΔDG*^ mice displayed a lack of LTP at perforant path–granule cell synapses in the DG, whereas LTP in the CA1 subfield was unaffected. This electrophysiological phenotype was associated with a very selective impairment in a rapid, flexible hippocampal memory system that may contribute to aspects of episodic memory performance in humans. These results demonstrate a clear dissociation between SWM and SRM on the radial maze following the manipulation of synaptic plasticity exclusively within the DG of the hippocampal formation.

## Abbreviations

AMPA: l-α-amino-3-hydroxy-5-methyl-4-isoxazelopropionate
Cre: cause of recombination enzyme
DG: dentate gyrus
DL-AP5: dl-2-amino-5-phosphonopentanoic acid
ES: embryonic stem
LTP: long-term potentiation
NMDAR: *N*-methyl-d-aspartate receptor
SRM: spatial reference memory
SWM: spatial working memory

## Supplementary material

The following supplementary material may be found on http://www.blackwell-synergy.com

Appendix S1. Assessment of sensimotor function and emotionality in *NR1^ΔDG^* mice.
