# Supplementary material for: Impaired spatial working memory but spared spatial reference memory following functional loss of NMDA receptors in the dentate gyrus
Source: Eur J Neurosci. 2007 Feb;25(3):837–46. doi: 10.1111/j.1460-9568.2007.05312.x (PMC2777262; doi:10.1111/j.1460-9568.2007.05312.x)
Supplement: Supplementary file 1 [file ejn0025-0837-SD1.doc]

#### Supplementary Material – “Impaired spatial working memory but spared spatial reference memory following functional loss of NMDA receptors in the Dentate Gyrus.”

B. Niewoehner^1^, F. Single^2^, ∅. Hvalby^3^, V. Jensen^3^, S. Meyer^2^, P.H. Seeburg^2^, J.N.P. Rawlins^1^, R. Sprengel^2^, D.M. Bannerman^1^

*Submitted to European Journal of Neuroscience*

# Supplementary Material - Behaviour

**Assessment of Sensorimotor Function and Emotionality in *NR1^ΔDG^* mice**

**Subjects.**

The same subjects were used throughout all behavioural testing, with all mice undergoing the full test battery to assess sensorimotor function and emotionality prior to commencing cognitive testing on the radial maze (see main text). The cohort consisted of male transgenic mice and litter-mates, comprising *NR1^ΔDG^* mice (n=8), heterozygous *NR1^2lox^/TG^LC1^* mice (n=12) and heterozygous *NR1^2lox^/TG^CN10-itTA^* mice (n=4). As the behavioural performance of the two groups of heterozygous mice were indistinguishable, their data were combined forming a single control group (Control; n=16). The order in which animals underwent assessment on the various tests was as follows: black/white alley, light/dark box (start in light), hyponeophagia test 1, spontaneous locomotor activity, accelerating rotarod, horizontal bar, multiple static rods, successive alleys (modified version of the elevated plus maze), light/dark box (start in dark) and hyponeophagia test 2. All experiments were conducted under the auspices of U.K. Home Office Project and Personal licences held by the authors.

**Methods.**

Detailed descriptions of the testing apparatus and methodologies have been provided elsewhere unless otherwise stated below (see Contet et al., 2001; Deacon et al., 2002a, Deacon et al., 2002b; Bannerman et al., 2004).

*Successive alleys test*

The successive alleys test is similar, in principle, to the elevated plus maze (see e.g. McHugh et al., 2004). The apparatus consisted of four successive wooden alleys (each 25 cm long), of increasing anxiogenic character. The less anxious the animal, the more it might be expected to venture out into the more exposed, open arms of the apparatus. Section or alley 1 was painted black, had 25 cm high walls, and was 8.5 cm wide. A 0.5 cm step down led to alley 2 which was again 8.5 cm wide, but had 1.3 cm high walls and was painted grey. A 1.0 cm step down led to alley 3 which was 3.5 cm wide, had 0.8 cm high walls and was white. A 0.4 cm step led down to alley 4 which was also white, 1.2 cm wide with 0.2 cm high walls. The apparatus was positioned on a table 0.7 m above the floor such that Section 1 was actually on the table, but Sections 2-4 extended out above the floor. The behaviour of the animals was assessed using a camera mounted directly above the apparatus, which was connected to a video recorder, allowing on- and off-line analysis by an observer in an adjacent room. Each mouse was brought into the room and then placed individually onto the maze in Section 1, facing away from sections 2-4. Latencies to cross into each section (all four paws), and the total times spent in each section were recorded. Trials lasted for 300 s. If the mouse fell from the maze it was immediately replaced at the junction between the section from which it fell and the preceding section.

*Light/Dark Box*

The apparatus consisted of an open, white compartment (the ‘light’ side; 30 x 20 x 20 cm) connected to a dark box which was painted black and covered with a lid (the ‘dark’ side; 15 x 20 x 20 cm). There was a 10 x 5 cm entrance between the two compartments. The aversiveness of the ‘light’ compartment was increased by additional illumination from a 60 W anglepoise lamp that was directed into the apparatus from 45 cm above the floor. One wall of the ‘light’ side was made of clear perspex, allowing the experimenter to view the mouse.

Each mouse was tested twice in the apparatus. On the first test the mouse was placed into the ‘light’ side, whereas on the second trial (conducted approximately one week later) the mouse was placed into the ‘dark’ side. The tests lasted for 5 min. Latency to first cross from one compartment to the other, and total time spent in each compartment were recorded on each occasion.

*Black/White alley*

In contrast to previously published methods, no grey habituation alley was used on this occasion. Mice were placed directly into the black section of the black/white alley.

*Hyponeophagia*

The first test of hyponeophagia assessed whether mice would drink 50% sweetened condensed milk on a white plexiglass base, covered by a transparent plastic jug as described previously (Bannerman et al., 2004). The second test assessed whether mice would eat 45 mg Noyes pellets (Formula A/I; P.J. Noyes, Lancaster, NH) on the exposed arm of an elevated T-maze. The T-maze was painted grey and was surrounded by a 10 cm wall. The mice were placed in one arm of the maze (10 x 20 cm) which was blocked off by a grey wooden block.

*Multiple static rods.*

Three rods of differing diameter were used to assess motor function in this study. The diameters of the rods that were used were 3.3, 2.1 and 0.8 cm.

*Data Analysis.*

Parametric data are presented as means (± s.e.m.), and were analysed by ANOVA. Where necessary, data were subjected to square root transformation prior to analysis. Significant interactions were further examined using analysis of simple main effects. Non-parametric data are presented as medians (± IQR), and were analysed using a non-parametric, Kruskal-Wallis ANOVA on ranks.

**Results.**

Control and *NR1^ΔDG^* mice were indistinguishable on the majority of tests of emotionality (see Table 1). The exception was performance on the successive alleys test (a modified form of the elevated plus maze) in which the *NR1^ΔDG^* mice were more inclined to venture into the more anxiogenic compartments (sections 2 and 3), suggesting that they were less anxious (see Table 2). There were no differences between the two groups of mice in terms of sensorimotor performance (see Table 3).

**Conclusions.**

In summary, although there was some suggestion from the successive alleys test that the *NR1^ΔDG^* mice were less anxious than controls, in general there were no clear differences between the two groups of mice in terms of emotionality or sensorimotor performance.

**Table Legends.**

*Table 1.* Comparison of control (n=16) and *NR1^ΔDG^* mice (n=8) on tests of emotionality (black/white alley, light/dark box and hyponeophagia). Parametric data are presented as means (± s.e.m.), and were analysed by ANOVA. Where necessary, data were subjected to square root transformation prior to analysis (*Hyponeophagia*). Non-parametric data are presented as medians (± IQR), and were analysed using a non-parametric, Kruskal-Wallis ANOVA on ranks (*Light/Dark Box Version 2*).

*Table 2.* Comparison of control (n=16) and *NR1^ΔDG^* mice (n=8) on the successive alleys test. Total times spent in each compartment (s) are presented as means (± s.e.m.). Analysis of time spent in the 4 alleys was conducted using a two way repeated measures ANOVA on the square root transformed data. Since the time spent in alley 4 is never independent of time spent in the other 3 alleys, the *p* values were adjusted to reflect a reduction in the degrees of freedom in the main effect of alley and the genotypes by alleys interaction. The ANOVA revealed a main effect of alley (F (2,66) = 119.5; p < 0.01) and a significant genotypes by alleys interaction (F (2,66) = 3.4; p < 0.05). The values in the table above (Statistics) correspond to the effect of group for each of the 4 alleys taken from a subsequent analysis of simple main effects. Latencies to enter compartments 2 and 3 are presented as medians (± IQR), and were analysed using a non-parametric, Kruskal-Wallis ANOVA on ranks. Latencies to enter compartment 4 are presented as means (± s.e.m.) and were analysed using ANOVA.

*Table 3.* Comparison of control (n=16) and *NR1^ΔDG^* mice (n=8) on tests of sensorimotor function (accelerating rotarod, horizontal bar, multiple static rods, spontaneous locomotor activity). Parametric data are presented as means (± s.e.m.), and were analysed by ANOVA. Where necessary, data were subjected to square root transformation prior to analysis (*Multiple static rods – orientation times*). Non-parametric data are presented as medians (± IQR), and were analysed using a non-parametric, Kruskal-Wallis ANOVA on ranks (*Horizontal bar*).

**References**

Bannerman DM, Deacon RMJ, Brady S, Bruce A, Sprengel R, Seeburg PH, Rawlins JNP (2004). A comparison of GluR-A deficient and wild type mice on a test battery assessing sensorimotor, affective and cognitive behaviors. *Behavioral Neuroscience,*118; 643-647*.*

Contet C, Rawlins JNP, Deacon RMJ (2001). A comparison of 129S2/SvHsd and C57BL/6JolaHsd mice on a test battery assessing sensorimotor, affective and cognitive behaviours: implications for the study of genetically modified mice. *Behavioural Brain Research,* 124; 33-46.

Deacon RMJ, Bannerman DM, Kirby BP, Croucher A, Rawlins JNP (2002a). The effects of cytotoxic hippocampal lesions in mice on a cognitive test battery. *Behavioural Brain Research,* 133; 57-68.

Deacon RMJ, Croucher A, Rawlins JNP (2002b). Hippocampal cytotoxic lesion effects on species-typical behaviours in mice. *Behavioural Brain Research,* 132; 203-213.

McHugh SB, Deacon RMJ, Rawlins JNP, Bannerman DM (2004). Amygdala and ventral hippocampal lesions contribute differentially to mechanisms of fear and anxiety. *Behavioral Neuroscience,* 118; 63-78.

**Supplementary Material – Electrophysiology**

The perforant path can be sub-divided into a lateral and medial component, named after their localized origin in the lateral and medial entorhinal cortex, respectively. The lateral perforant path (lpp) makes excitatory contacts onto dentate granule cell dendrites in the outer third, whereas the medial perforant path (mpp) forms excitatory synapses in the middle third of the molecular layer (Hjorth-Simonsen, 1972; Hjorth-Simonsen & Jeune, 1972). Physiologically, these two pathways can be distinguished in slices, because lpp/granule cell synapses show paired-pulse facilitation, whereas mpp/granule cell synapses exhibit paired-pulse depression (McNaughton, 1980).

*Preparation of slices*

Experiments were performed on hippocampal slices prepared from adult (2-4 months old) *NR1^ΔDG^* mice and wild type control mice. The animals were sacrificed with desflurane (Suprane, Baxter AS, Oslo, Norway) and the brains were removed and cooled in artificial cerebrospinal fluid (ACSF, 0-4^o^C, bubbled with 95% O_2_ - 5% CO_2_, pH 7.4) containing (in mM): 124 NaCl, 2 KCl, 1.25 KH_2_PO_4_, 2 MgSO_4_, 1 CaCl_2_, 26 NaHCO_3_ and 12 glucose. Transverse slices (400 μm) were cut from the middle portion of each hippocampus with a vibroslicer and placed in a humidified interface chamber at 30 + 1 ^o^C and perfused with ACSF containing 2 mM CaCl_2_. In order to enhance the induction of LTP in the dentate gyrus, we partially blocked GABA_A_–mediated inhibition with (-)-bicuculline methochloride (6μM; Tocris Cookson Ltd., Bristol, UK). The resulting hyperexcitability was counteracted by increasing the concentration of Ca^2+^ and Mg^2+^ to 4mM in accordance with earlier reports (Wigström & Gustafsson, 1983; 1985). In some experiments 50μM DL-2-amino-5-phosphonopentanoic acid (DL-AP5; Sigma-Aldrich, Oslo, Norway) was present during the experiments in order to block NMDA receptor-mediated synaptic plasticity.

*Stimulation and recordings*

Orthodromic synaptic stimuli (50 μs, < 300μA, 0.1 Hz) were delivered alternately through two tungsten electrodes, either with one situated in the stratum radiatum and another in the stratum oriens of the CA1 region, or with electrodes in the outer and middle molecular layer of the upper blade of the dentate area. Extracellular synaptic responses were monitored by two glass electrodes (filled with ACSF) placed in the corresponding synaptic layers. After obtaining stable synaptic responses in both pathways (0.1 Hz stimulation) for at least 10-15 minutes, one of the pathways was tetanized (100 Hz, 1 s) whereas the other served as an non-tetanized control pathway. The tetanic stimulation strength was just above the threshold for generation of a population spike in response to a single test stimulus.

*Analysis*

We assessed synaptic efficacy by measuring the slope of the fEPSP in the middle third of its rising phase. Six consecutive responses (1 min) were averaged and normalized to the mean value recorded 1-4 min prior to tetanic stimulation. Data were pooled across animals of the same genotype and are presented as mean ± s.e.m. The difference between tetanized and non-tetanized pathways was statistically evaluated by a student’s paired, two-tailed *t* test, and when comparing LTP levels between control and *NR1^ΔDG^* mice we used a linear mixed model analysis.

*NMDA receptor-dependency of LTP in dentate gyrus and CA1.*

As expected, the NMDA receptor blocker DL-AP5 (50μM) produced a block of tetanus-induced LTP in slices from wild-type mice, in both the lpp/granule cell synapses (1.02 + 0.04 versus 0.98 + 0.04 n = 8; *P* = 0.92; data not shown) and mpp/granule cell synapses (1.02 + 0.11 versus 1.03 + 0.02 n = 8; *P* = 0.23; data not shown), thus underlining NMDA receptor involvement in LTP at these synapses (Hanse & Gustafsson, 1992). The presence of DL-AP5 (50μM) also completely blocked CA1 LTP in slices from wild type animals (tetanized versus control pathway: 1.00 + 0.03 versus 1.05 + 0.04, n = 8; *P* = 0.65; data not shown).

Hanse, E. & Gustafsson, B. Long-term potentiation and field EPSPs in the lateral and medial perforant paths in the dentate gyrus *in vitro*: a comparison. *Eur. J. Neurosci.* **4,** 1191-1201 (1992).

Hjorth-Simonsen, A. Projection of the lateral part of the entorhinal area to the hippocampus and fascia dentata. *J. Comp. Neurol.* **146,** 219-232 (1972).

Hjorth-Simonsen, A. & Jeune, B. Origin and termination of the hippocampal perforant path in the rat studied by silver impregnation. *J. Comp. Neurol.* **144,** 215-232 (1972).

McNaughton, B.L. Evidence for two physiologically distinct perforant pathways to the fascia dentata. *Brain Res.* **199,** 1-19 (1980).

Wigström, H. & Gustafsson, B. Large long-lasting potentiation in the dentate gyrus *in vitro* during blockade of inhibition. *Brain Res.* **275,** 153-158 (1983).

Wigström, H. & Gustafsson, B. Facilitation of hippocampal long-lasting potentiation by GABA antagonists. *Acta Physiol. Scand.* **125,** 159-172 (1985).

| Task and Measure | Control | *NR1^ΔDG^* | Statistics |
| --- | --- | --- | --- |
| Black/White Alley | | | |
| Latency to enter white compartment (s) | 23.6 ± 2.6 | 25.6 ± 7.0 | F < 1; P > 0.20 |
| Time spent in white compartment (s) | 34.0 ± 2.6 | 41.8 ± 3.2 | F (1,22) = 3.1; P > 0.05 |
| Light/Dark Box Version 1 (Start in light compartment) | | | |
| Latency to cross (s) | 21.2 ± 3.7 | 17.0 ± 5.0 | F < 1; P > 0.20 |
| Time spent in light compartment (s) | 104.7 ± 11.0 | 107.8 ± 24.1 | F < 1; P > 0.20 |
| Light/Dark Box Version 2 (Start in black compartment) | | | |
| Latency to cross (s) | 11.5 (7.0 – 41.5) | 18.5 (5.5 – 86.0) | H < 1; P > 0.20 |
| Time spent in light compartment (s) | 78.0 (45.0 – 96.0) | 105.0 (42.5 – 212.0) | H < 1; P > 0.20 |
| *Hyponeophagia* | | | |
| Mean latency to consume food (s) | 65.6 ± 14.4 | 67.6 ± 20.4 | F < 1; P > 0.20 for square root transformed data |

TABLE 1. Tests of Emotionality (1)

| Task and Measure | Control | *NR1^ΔDG^* | Statistics |
| --- | --- | --- | --- |
| Total time per compartment (s) ^1^ | | | |
| Compartment 1 | 226.6 ± 7.7 | 183.3 ± 22.9 | F (1,88) = 3.5; 0.05 < P < 0.10 |
| Compartment 2 | 43.5 ± 4.7 | 70.3 ± 13.3 | F (1,88) = 4.1; P < 0.05 |
| Compartment 3 | 15.5 ± 2.9 | 30.8 ± 8.9 | F (1,88) = 4.0; P = 0.05 |
| Compartment 4 | 14.4 ± 3.1 | 15.8 ± 5.4 | F < 1; P > 0.20 |
| *Latency to enter (s)* | | | |
| Compartment 2 | 12.0 (8.0 – 25.5) | 6.0 (4.0 – 7.5) | H (1) = 7.6; P < 0.01 |
| Compartment 3 | 139.5 (21.0 – 270.5) | 180.5 (44.0 – 268.0) | H < 1.0; P > 0.20 |
| Compartment 4 | 166.1 ± 29.2 | 179.4 ± 42.9 | F < 1; P > 0.20 |

**TABLE 2. Tests of Emotionality (2) – The Successive Alleys Test (modified form of elevated plus maze).**

*^1^* Analysis of time spent in the 4 alleys was conducted using a two way repeated measures ANOVA on the square root transformed data. Since the time spent in alley 4 is never independent of time spent in the other 3 alleys, the *p* values were adjusted to reflect a reduction in the degrees of freedom in the main effect of alley and the genotypes by alleys interaction. The ANOVA revealed a main effect of alley (F (2,66) = 119.5; p < 0.01) and a significant genotypes by alleys interaction (F (2,66) = 3.4; p < 0.05). The values in the table above (Statistics) correspond to the effect of group for each of the 4 alleys taken from a subsequent analysis of simple main effects.

| Task and Measure | Control | *NR1^ΔDG^* | Statistics |
| --- | --- | --- | --- |
| Rotarod | | | |
| Speed at fall (rpm) | 10.7 ± 1.1 | 9.5 ± 2.4 | F < 1; P > 0.20 |
| *Horizontal Bar* | | | |
| Ranked score | 5.0 (5.0 – 5.0) | 5.0 (4.0 – 5.0) | H < 1; P > 0.20 |
| *Multiple Static Rods* | | | |
| Mean orientation time (s) | 49.7 ± 9.4 | 59.5 ± 13.3 | F < 1; P > 0.20 for main effect of group and interaction for square root transformed data |
| Mean transit time (s) | 61.7 ± 9.7 | 73.7 ± 13.7 | F < 1; P > 0.20 for main effect of group and interaction |
| *Spontaneous Locomotor Activity* | | | |
| Mean total beam breaks in 2 hours | 1768.0 ± 206.2 | 2086.9 ± 350.5 | F < 1; P > 0.20 |

TABLE 3. Tests of Sensorimotor Function.
